# Bio-objects and their Boundaries: Governing Matters at the Intersection of Society, Politics, and Science

**DOI:** 10.3325/cmj.2011.52.648

**Published:** 2011-10

**Authors:** Ingrid Metzler, Andrew Webster

After many hours spent exploring Web sites of clinics and discussing with other patients across the globe, a Swedish couple decides the only way to treat their child’s disease is to venture to China to gain access to new stem cell therapies not available in their home country. At the same time a Swedish researcher, who is increasingly concerned by the flourishing of promissory and yet unproven stem cell therapies around the globe, joins forces with British researchers to draw on stem cell lines derived from an in vitro fertilization (IVF) embryo to explore the mechanisms of this very disease. Yet another group of British researchers tinkers with transgenic mice to achieve a similar aim (research that is co-sponsored by a US-American patient organization, but that a trans-national group of animal rights activists finds deeply worrisome). Roughly a thousand miles toward the South, an Italian woman in her early 40s makes arrangements with a fertility center to be able to draw on egg cells that had been donated by another woman, an arrangement that she makes over phone and in English with a Ukrainian fertility center as egg donation is not legally available in her home country and offered at prices that she cannot afford in countries closer to her home. Detached from these locations and spaces and yet somehow connected, policy-makers in Brussels ponder over how to integrate these various promissory objects and concerned subjects into European innovation policies, to make sure that Europe does not miss the opportunity that stem cells offer toward a knowledge-based future.

These instances take place in different spaces and they involve very different subjects and objects, as well as a range of novel biological forms that cannot be easily classified along conventional categories. Yet, what might we learn if we cease to treat them as different phenomena? What might we learn from bringing these different objects together and explore their similarities and differences? And how might the knowledge generated through such a convergence enhance both our understanding of how we know and make sense of our world as well as how we approach and choose to live in it? Most importantly, they deal with the issue of “life” and raise questions about how life enters into the picture. Doctors confront such an issue routinely through their engagement with reproductive medicine and its converse, dealing with the end of life itself when managing death. But our story above suggests that “life” is entering the picture in new ways and crossing boundaries – between human and human, human and animal, the natural and unnatural, and between the very meaning of life and death.

Questions of such kinds inform the recently established international research network, “Bio-objects and their Boundaries: Governing Matters at the Intersection of Society, Science and Politics,” launched in December 2010. This “Action”, funded by the European Cooperation in Science and Technology (COST) framework, comprises a very heterogeneous group of young and more experienced researchers from a variety of disciplines and fields of inquiry, ranging from science studies, philosophy, through to the life sciences, a network under the formal oversight of the co-authors (Box 1) (1).

Box 1 **European Cooperation in Science and Technology (COST) Action IS1001: “Bio-objects and their Boundaries: Governing Matters at the Intersection of Society, Politics, and Science”**

• This Action, a research network, is funded by COST.

• It was launched in December 2010 and is scheduled to run until December 2014.

• Web site: *http://www.bioobjects.eu.*

All members engage with bio-medical and bio-technological innovation processes in their daily lives as researchers in our home institutions. For instance, some of us work with human embryonic stem cell (hESC) lines on benches in laboratories, others venture into cross-country comparisons of the regulatory settlements of hESC research, while still others follow hESCs and hESC scientists in their daily work on benches and desk tops to get a better sense of what is going on. In this Action, all of us bring our individual experiences, insights and findings, as well as questions and puzzles, in order to share them, exchange them, and to learn from this synergy. Indeed, a central tenet of this collaborative endeavor is the understanding that while discrete bio-medical and bio-technological innovation processes and their products have different effects and implications in different spaces and domains, much can be learnt if we overcome disciplinary boundaries, and try to assemble these different objects – as well as the researchers that conduct research on them or that help make them in the first place. We venture into such an endeavor through the concept of “bio-objects.”

## Bio-objects and bio-objectification processes

From a descriptive perspective, bio-objects are meant to refer to, first, creatures that have been made at the work benches of the life sciences, such as genetically-modified organisms or transpecies animals, as well as, second, entities that we are, by now, much more familiar with but that have been brought into new spaces, such as stem cells that are removed from the cord blood after deliveries and stored in cord blood banks, IVF embryos that dwell in Petri dishes in laboratories, or human tissue samples that have been cut from a patient’s body, frozen for storage and kept in a tissue repository, together with her clinical records and life-style information. For sure, all these instances of bio-objects are distinct in biological terms, and they come with their own histories and challenges, and yet they nonetheless share a number of similarities.

First, they are the products of various efforts to know and enhance (human) life – that is *bio(s)–*, through intervening in and objectifying it, that is through creating often very tangible objects that can be leveraged and stored, as well as circulated and exchanged. Such objects might be the product of efforts to redirect living processes in laboratories; yet, they might also take a less “wet” shape – such as in aggregation of numbers of patients and research subjects in clinical studies. Second, they tend to be characterized by ever-greater fluidity and mobility across different domains. This means that a bio-object associated with biomedical research may find its way into the food system or the environment or become part of a repository (as in biobanks). Third, they tend to disrupt the conventional boundaries and identities of biological forms and categories, such as the boundaries between human and animals or between the natural and artificial, sitting ambiguously in between those entities that we tend to conceptualize as human subjects and as non-human objects instead, sometimes troubling or even unsettling this very distinction. Last but not least, they tend to trigger very different and sometimes contrasting demands. They are often imbued with hopes, promising to enhance our knowledge on health and disease, to regenerate body parts or, sometimes, ailing economies, rendering our collective life safer, healthier, and more productive (2,3). They are closely tied to ways in which European identity is imagined at the beginning of the 21st century. Yet, bio-objects also trigger a range of concerns. Such concerns range from citizens who are upset by the “objectification” of IVF embryos or concerned by the trading of egg cells, to policy makers who might not be concerned by the proliferation and circulation of these objects but by their uneven paths of development and by the difficulties and uncertainties in which some of these ambiguous objects are entangled in when they are moved to clinics or the markets. This was evident, for example, during the recent debate in Europe over how to classify tissue-engineered products or therapies that involve the manipulation and reconfiguring of cells – was the result to be a device, a drug, a medicine? At one point, the European Medicines Agency had over 25 competing definitions, a situation only resolved by the creation of the term “Advanced Therapy Medicinal Products,” a concept found nowhere else in the regulatory world.

These features imply that it is not very instructive to conceptualize bio-objects as stable entities. Rather, we should see them as “boundary crawlers” (4) that move and are moved across different domains, engendering different and sometimes conflicting demands, and as always only the preliminary product of ongoing processes to tame life and to render it more productive. This is why we do not take them as in some way “given,” a pre-defined class of entities; instead, we find it more useful to explore them as a result of “bio-objectification” processes, that is those technological interventions and labors through which we seek to tame or control life but typically in doing so render it more open to contestation, located in new spaces of significance. How this works out in practice in different spaces, is the major question that we seek to tackle in our collaborative research effort.

## Boundaries, governance, and generative relations

In our Action, we explore bio-objects and bio-objectification processes along three axes – each of which is organized into a “working group” that draws on members’ current research and disciplinary expertise to explore a range of substantive issues within a specific domain. Working group 1 explores boundary changes between human and non-human and living and non-living. Working group 2 compares the modes in which bio-objects are governed at different levels, ranging from efforts at a global scale down to nation states and their institutions as more traditional sites of governance to practices of self-governance of individuals such as patients. Working Group 3 seeks to explore what we conceptualize as “generative relations,” that is those economic, political, or social contexts that facilitate bio-objectification processes, and which – in turn – are themselves shaped or reified through such processes (Box 2).

Box 2 **Working Groups**

• Working group 1: Boundary changes between human/non-human and living/non-living Working group 1 examines the changing boundaries of the human, nonhuman, and society with the emergence of new and changing bio-objects.

• Working group 2: The governance of bio-objects The second Working Group examines the governance of new bio-objects, and the socio-cultural and political regulations involved in the boundary shifts that they bring about.

• Working group 3: Bio-objects and their “generative relations.” The third working group explores the emergence of new kinds of social and economic relations prompted by processes of “bio-objectification.”

The objectives of our collaborative effort are, first, to develop a conceptual repertoire that helps us grasp these processes and that is also helpful for life scientists and biomedical researchers as well as informative for policy makers. A second objective of this Action relates to network building (Box 3). Indeed, a consistent part of our network consists of scholars who have participated in a Marie Curie Training Programme at the Science and Technology Studies Unit at the Department of York. In the meantime, the launch of this Action has helped us enrich this network with other young scholars, as well as more experienced ones, and new participants are welcome to attend one of our public events, or to join one of our working groups. In the meantime, readers will have the opportunity to learn more on this Action in the forthcoming issues of this journal.

Box 3 **Objectives**

• The first objective of this Action is to improve our understanding of processes of new scientific and technological development, in particular the making, trading, and socio-cultural use of new bio-objects emerging at present in Europe, in order to strengthen Europe’s capacity to both exploit and manage the intended and unintended effects of these processes.

• A second objective relates to capacity building, aiming at the building of a strong network that unites young researchers and more experienced ones with different disciplinary backgrounds from different European countries.
